# No association between a candidate *TCF7L2 *variant and risk of breast or ovarian cancer

**DOI:** 10.1186/1471-2407-9-312

**Published:** 2009-09-04

**Authors:** Ellen L Goode, Csilla Szabo, Ludmila Prokunina-Olsson, Robert A Vierkant, Zachary S Fredericksen, Francis S Collins, Kristin L White, Michele Schmidt, Brooke L Fridley, Fergus J Couch

**Affiliations:** 1 Health Sciences Research, Mayo Clinic College of Medicine, Rochester, USA; 2 Laboratory of Translational Genomics, Division of Cancer Epidemiology and Genetics, National Cancer Institute, National Institutes of Health, Bethesda, USA; 3 Office of the Director, National Institutes of Health, Bethesda, USA; 4 Laboratory Medicine and Pathology, Mayo Clinic College of Medicine, Rochester, USA

## Abstract

**Background:**

TCF7L2 is a transcription factor involved in Wnt/β-catenin signaling which has a variant known to be associated with risk of Type 2 diabetes and, in some studies, with risk of certain cancers, including familial breast cancer. No studies of ovarian cancer have been reported to date.

**Methods:**

Two clinic-based case-control studies at the Mayo Clinic were assessed including 798 breast cancer cases, 843 breast cancer controls, 391 ovarian cancer cases, and 458 ovarian cancer controls. Genotyping at *TCF7L2 *rs12255372 used a 5' endonuclease assay, and statistical analysis used logistic regression among participants as a whole and among *a priori*-defined subsets.

**Results:**

No associations with risk of breast or ovarian cancer were observed (ordinal model, p = 0.62 and p = 0.75, respectively). In addition, no associations were observed among sub-groups defined by age, BMI, family history, stage, grade, histology, or tumor behavior.

**Conclusion:**

Although the biology of the Wnt/β-catenin signaling pathway and prior association between rs12255372 and numerous phenotypes warranted examination of this *TCF7L2 *SNP, no compelling evidence for association with breast or ovarian cancer was observed.

## Background

Transcription factor 7-like 2 (*TCF7L2*) encodes a transcription factor involved in Wnt/β-catenin signaling pathway which encompasses an intronic single-nucleotide polymorphism (SNP, rs12255372) that has been associated with risk of Type 2 diabetes in linkage studies and genome-wide association studies [1-4] with potential modification by obesity [5]. TCF7L2 forms an active nuclear complex with β-catenin that binds and induces the expression of target genes involved in cellular proliferation, evasion of apoptosis, and tissue invasion and metastasis. Because of the protein's relevance in this canonical cancer pathway, several cancer association studies have been conducted. Studies report associations with increased risk of familial breast cancer [6] and aggressiveness of prostate cancer [7]; there are conflicting reports in colon cancer showing decreased risk [8], increased risk [9], or differential risk by NSAIDs use [10]. We sought to assess the role of this polymorphism in risk of breast and ovarian cancer and, based on other reports, in subsets defined by body mass index (BMI), family history, and measures of disease aggressiveness (e.g., stage, histological subtypes).

## Methods

Female participants were recruited at Mayo Clinic in Rochester, MN. Invasive breast cancer cases (N = 798) were over 18 years of age and enrolled within six months of diagnosis. Invasive or borderline epithelial ovarian cancer cases (N = 391) were over 20 years of age and living in the Upper Midwest enrolled within one year of diagnosis. Controls (breast cancer N = 843; ovarian cancer N = 458) were frequency-matched on age and region of residence and seen for general medical examinations. Risk factor information was collected through interviews, clinical data was abstracted from medical records, and DNA was extracted from peripheral blood (Gentra). All breast cancer participants and 98% of ovarian cancer participants were white non-Hispanic. Details are provided elsewhere [11,12].

Genotyping at *TCF7L2 *rs12255372 was performed on 384-well plates using TaqMan™ (Applied Biosystems). For breast and ovarian cancer, respectively, call rates were high (98.1%, 95.9%), genotypes were in Hardy-Weinberg equilibrium (p = 0.23, p = 0.96), white non-Hispanic control minor allele frequencies were as expected (27.6%, 26.0%), and concordance across duplicates was 100%. Logistic regression in SAS v. 8 (SAS Institute) was used to estimate odds ratios (ORs) and 95% confidence intervals (CIs), primarily assuming an ordinal effect with simple tests for trend; heterozygous and minor allele homozygous ORs were also estimated. Ovarian cancer analyses were adjusted for age and region of residence; breast cancer analyses were additionally adjusted for oral contraceptive use, hormone therapy use, age at first birth, age at menarche, menopausal status, and smoking pack years. Stratified analyses were performed on sub-groups of menopausal status, BMI, family history, and estrogen, progesterone, and HER2 receptor status (breast cancer) and age, family history, BMI, stage, grade, histology, and tumor behavior (ovarian cancer).

## Results

For both breast and ovarian cancer, no overall association with risk was observed (per-allele OR, 95% CI; breast 1.04, 0.89-1.22; ovarian 0.96, 0.77-1.20). Breast cancer stratified analyses revealed no associations (p's > 0.05) among groups defined by menopausal status, BMI, family history, or tumor histology (Table 1). Assuming independent genotype effects (co-dominance), differential risk of breast cancer by genotype for HER2 positive was suggested (GT vs GG 1.68, 1.12-2.53; TT vs GG 0.49, 0.17-1.45; 2 d.f. p = 0.01). Exploratory analyses assuming dominance (GT/TT v GG) suggested elevated breast cancer risks for pre-menopausal (1.46, 0.99-2.15; p = 0.06), HER2 positive (1.48, 1.00-2.01; p = 0.05), and triple negative cases (2.01, 1.10-3.67; p = 0.02). Although subgroups were limited in size, these results are consistent with involvement of rs12255372 in non-luminal tumors. Analyses among ovarian cancer sub-groups did not suggest varied associations by age, BMI, family history, stage, grade, histology, or tumor behavior (Table 2). A significant association (p = 0.02) among 12 cases with grade 1 disease compared to 458 controls is likely due to chance, as the number of strata examined was large, sample size was small, no trend with grade was seen, and no biological explanation fits this observation.

**Table 1:** *TCF7L2 *rs12255372 and risk of breast cancer

		N	N	Ordinal OR (95% CI)		Co-Dominant ORs (95% CI)		
				
		Cases	Controls	Per T-allele	P (trend)	GT v GG	TT v GG	P (2 d.f.)
Total	---	779	830	1.04 (0.89-1.22)	0.62	1.16 (0.93-1.43)	0.92 (0.62-1.36)	0.31
Menopausal	Pre	268	213	1.28 (0.94-1.75)	0.12	1.51 (1.01-2.26)	1.21 (0.56-2.61)	0.14
status	Post	467	573	0.95 (0.77-1.16)	0.59	1.03 (0.78-1.34)	0.79 (0.49-1.29)	0.60
BMI	≤ 25.9	341	394	1.07 (0.84-1.37)	0.59	1.25 (0.90-1.73)	0.91 (0.50-1.64)	0.34
	> 25.9	406	393	1.03 (0.82-1.30)	0.78	1.13 (0.83-1.54)	0.92 (0.52-1.63)	0.66
Family	No	424	480	1.04 (0.84-1.30)	0.70	1.09 (0.81-1.46)	1.02 (0.60-1.73)	0.85
history	Yes	336	312	0.99 (0.77-1.28)	0.95	1.25 (0.89-1.76)	0.68 (0.36-1.28)	0.14
Estrogen	Positive	472	830	1.09 (0.91-1.31)	0.34	1.16 (0.90-1.49)	1.10 (0.70-1.71)	0.50
Receptor (ER)	Negative	107	830	1.11 (0.81-1.52)	0.52	1.41 (0.92-2.17)	0.84 (0.36-1.96)	0.21
Progesterone	Positive	429	830	1.21 (0.93-1.35)	0.23	1.19 (0.92-1.54)	1.15 (0.73-1.81)	0.39
Receptor (PR)	Negative	147	830	1.00 (0.76-1.32)	0.99	1.19 (0.82-1.71)	0.74 (0.35-1.56)	0.40
HER2 status	Positive	122	830	1.14 (0.83-1.55)	0.42	**1.68 (1.12-2.53)**	**0.49 (0.17-1.45)**	**0.01**
	Negative	269	830	1.02 (0.82-1.27)	0.85	1.04 (0.78-1.40)	1.01 (0.60-1.70)	0.96
ER, PR, HER2	All Negative	51	842	1.47 (0.95-2.26)	0.08	2.16 (1.16-4.01)	1.36 (0.43-4.30)	0.05

BMI, body mass index, categorized by median value; family history, first-degree or second-degree family history; analyses adjusted for age, region of residence, oral contraceptive use, hormone therapy use, age at first birth, age at menarche, menopausal status (unless used as stratifiying variable), and pack years of smoking; n.a., not estimatable, bold indicates p < 0.05.

**Table 2:** *TCF7L2 *rs12255372 and risk of ovarian cancer

		N	N	Ordinal OR (95% CI)		Co-Dominant ORs (95% CI)		
				
		Cases	Controls	Per T-allele	P (trend)	GT v GG	TT v GG	P (2 d.f.)
Total	---	391	458	0.96 (0.77-1.20)	0.75	0.95 (0.71-1.26)	0.97 (0.56-1.68)	0.93
Age	< 60	191	206	1.15 (0.83-1.59)	0.40	1.04 (0.68-1.58)	1.60 (0.70-3.69)	0.54
	60+	200	252	0.82 (0.60-1.10)	0.19	0.87 (0.58-1.28)	0.60 (0.28-1.28)	0.38
BMI	≤ 26.2	164	226	1.00 (0.72-1.39)	1.00	0.96 (0.62-1.47)	1.08 (0.47-2.44)	0.96
	> 26.2	211	203	0.94 (0.69-1.30)	0.74	0.98 (0.65-1.49)	0.84 (0.38-1.84)	0.91
Family	No	329	403	0.93 (0.74-1.18)	0.58	1.01 (0.74-1.37)	0.77 (0.43-1.38)	0.66
history	Yes	50	32	2.18 (0.89-5.34)	0.09	1.20 (0.40-3.61)	21.1 (1.34-334)	0.09
Stage	1	103	458	1.00 (0.70-1.41)	0.99	0.95 (0.60-1.50)	1.08 (0.44-2.63)	0.96
	2	29	458	1.24 (0.65-2.35)	0.51	1.09 (0.49-2.41)	2.25 (0.28-17.8)	0.74
	3	196	458	0.86 (0.66-1.13)	0.28	0.82 (0.58-1.18)	0.80 (0.41-1.56)	0.52
	4	55	458	1.26 (0.78-2.02)	0.35	1.54 (0.82-2.89)	1.07 (0.35-3.26)	0.40
Grade	0	64	458	1.26 (0.80-1.99)	0.32	1.12 (0.64-1.95)	2.34 (0.52-10.4)	0.52
	1	12	458	**0.37 (0.16-0.88)**	**0.02**	0.19 (0.04-0.94)	0.15 (0.02-1.11)	0.09
	2	41	458	0.88 (0.53-1.46)	0.62	1.09 (0.54-2.23)	0.61 (0.21-1.81)	0.60
	3	146	458	0.99 (0.73-1.35)	0.94	0.97 (0.65-1.44)	1.02 (0.46-2.27)	0.98
	4	121	458	0.93 (0.68-1.29)	0.68	0.93 (0.60-1.42)	0.89 (0.40-1.98)	0.92
Histology	Serous	232	458	0.93 (0.72-1.21)	0.60	0.87 (0.62-1.21)	1.00 (0.52-1.93)	0.70
	Mucinous	27	458	1.24 (0.63-2.44)	0.53	0.89 (0.40-1.97)	n.a.	0.96
	Endometriod	64	458	0.94 (0.62-1.41)	0.76	1.55 (0.84-2.86)	0.53 (0.23-1.22)	0.07
	Clear cell	25	458	1.04 (0.54-2.00)	0.91	0.81 (0.35-1.90)	1.91 (0.23-15.7)	0.70
	Mixed cell	32	458	1.42 (0.76-2.68)	0.27	1.30 (0.60-2.80)	2.73 (0.35-21.4)	0.55
Behavior	Malignant	327	458	0.93 (0.74-1.17)	0.52	0.92 (0.68-1.25)	0.87 (0.49-1.53)	0.81
	Borderline	64	458	1.20 (0.76-1.88)	0.43	1.03 (0.59-1.80)	2.29 (0.52-10.2)	0.55

BMI, body mass index, categorized by median value; family history, first-degree or second-degree family history; analyses adjusted for age (unless used as stratifiying variable) and region of residence; n.a., not estimatable, bold indicates p < 0.05.

## Discussion

*TCF7L2 *has received much recent attention, as a consistently-replicated association that originated out of a traditional genetic linkage scan [3] that also appears consistently as a "top hit" for several phenotypes in genome-wide association studies [13]. Although association between rs12255372 and risk of Type 2 diabetes is thought to be due to impairment of insulin secretion [14], *TCF7L2 *has also long been known to have a role in Wnt/β-catenin signaling. Deregulation of the Wnt pathway is involved in the mechanisms of carcinogenesis [15], and mutations in Wnt-related genes have been detected in many cancers [16-18], indicating somatic mutations are important deregulation mechanisms of Wnt signaling. In breast cancer, we recently demonstrated that SNPs in the *AXIN2 *and *APC *genes are associated with risk [11]. In ovarian cancer, somatic mutations in *CTNNB1 *(encoding *β*-catenin) are directly linked to carcinogenic transformation, but they are rare and mostly found in endometrioid adenocarcinomas [19]. Ovarian expression studies show differences for distinct cellular components of the Wnt pathway between normal and cancer cells [20] implicating Wnt signaling in the molecular events leading to ovarian cancer despite the fact that gene mutations are uncommon [21].

Because of the importance of Wnt/β-catenin signaling in breast and ovarian cancer, TCF7L2's critical role in combining with β-catenin, clear associations of rs12255372 with non-cancer phenotypes, and suggestive evidence of association with cancer phenotypes, we hypothesized that genotypes at this SNP may be associated with risk of breast and/or ovarian cancer. However, no strong association was suggested in our analysis of almost 800 breast cancer cases and 850 controls, and almost 400 ovarian cancer cases and 450 controls.

These results contrast with a smaller analysis of 592 familial breast cancer cases and 735 controls which showed a per-allele OR of 1.19 (95% CI 1.01-1.42) [6]. Our breast cancer case-control series provided 80% power to detect an OR as low as 1.22, assuming a minor allele frequency of 0.25 and a Type I error rate of 0.05 and was adequately powered to detect similar modest associations. Additionally, there may be an association in smaller subsets consistent with non-luminal tumors. No ovarian cancer analyses have been reported to date. Previous reports in other traits suggested varied association by BMI, family history, and tumor features; although subset sample sizes were small, we found no evidence for association between rs12255372 and risk in any subset.

## Conclusion

Despite strong prior evidence that this SNP may play a role women's cancer susceptibility, we conclude that variation in *TCF7L2 *is not likely to be associated with risk of non-familial breast cancer or ovarian cancer. Because very modest associations could not be definitively ruled out and because increased risk may exist for triple-negative breast cancer cases, much larger studies will be required for more precise estimation of rs12255372's role in cancer susceptibility. In addition, given the role of Wnt/β-catenin signaling in cancer, identified associations of SNPs in the *AXIN2 *and *APC *genes with breast cancer and the increased risk conferred by family history for these conditions even after accounting for known syndromes, additional SNPs and genes within this pathway warrant further study.

## Competing interests

The authors declare that they have no competing interests.

## Authors' contributions

ELG drafted the manuscript and oversaw the ovarian cancer study; CS initiated the collaboration and guided subset analysis; LP-O provided preliminary results and interpreted results; RAV supervised ovarian cancer analysis; ZSF performed breast cancer analysis; FSC provided key interpretive insight; KLW compiled results and performed literature review; MS performed ovarian cancer analysis; BLF oversaw ovarian cancer analysis; and FJC oversaw the breast cancer study.

## Pre-publication history

The pre-publication history for this paper can be accessed here:

http://www.biomedcentral.com/1471-2407/9/312/prepub
